# Influence of dyskalemia at admission and early dyskalemia correction on survival and cardiac events of critically ill patients

**DOI:** 10.1186/s13054-019-2679-z

**Published:** 2019-12-19

**Authors:** Lila Bouadma, Stefan Mankikian, Michael Darmon, Laurent Argaud, Camille Vinclair, Shidasp Siami, Maité Garrouste-Orgeas, Laurent Papazian, Yves Cohen, Guillaume Marcotte, Lenka Styfalova, Jean Reignier, Alexandre Lautrette, Carole Schwebel, Jean-Francois Timsit, on behalf of the OUTCOMEREA STUDY GROUP, Jean-François Timsit, Elie Azoulay, Maïté Garrouste-Orgeas, Jean-Ralph Zahar, Christophe Adrie, Michael Darmon, Christophe Clec’h, Corinne Alberti, Adrien Français, Aurélien Vesin, Stephane Ruckly, Sébastien Bailly, Frederik Lecorre, Didier Nakache, Aurélien Vannieuwenhuyze, Carole Agasse, Bernard Allaouchiche, Olivier Andremont, Pascal Andreu, Laurent Argaud, Claire Ara Somohano, Francois Barbier, Jean-Pierre Bedos, Jérome Bedel, Julien Bohé, Lila Bouadma, Noel Brule, Cédirc Brétonnière, Christine Cheval, Elisabeth Coupez, Etienne de Montmollin, Loa Dopeux, Anne-Sylvie Dumenil, Claire Dupuis, Jean-Marc Forel, Marc Gainnier, Charlotte Garret, Antoine Gros, Akim Haouache, Romain Hernu, Samir Jamali, Sébastien Jochmans, Jean-Baptiste Joffredo, Hatem Khallel, Guillaume Lacave, Alexandre Lautrette, Virgine Lemiale, Mathilde Lermuzeaux, Guillaume Marcotte, Eric Le Miere, Jordane Lebut, Maxime Lugosi, Eric Magalhaes, Sibylle Merceron, Bruno Mourvillier, Benoît Misset, Delphine Moreau, Mathild Neuville, Laurent Nicolet, Laurent Papazian, Benjamin Planquette, Jean-Pierre Quenot, Aguila Radjou, Romain Sonneville, Jean Reignier, Bertrand Souweine, Carole Schwebel, Roland Smonig, Gilles Troché, Marie Thuong, Guillaume Thierry, Dany Toledano, Guillaume Van Der Meersch, Marion Venot, Olivier Zambon, Julien Fournier, Caroline Tournegros, Stéphanie Bagur, Mireille Adda, Vanessa Vindrieux, Sylvie de la Salle, Loic Ferrand, Stéphane Guessens, Helene Merle, Nadira Kaddour, Boris Berthe, Samir Bekkhouche, Kaouttar Mellouk, Mélaine Lebrazic, Carole Ouisse, Diane Maugars, Sylvie Conrozier, Igor Theodose, Manal Nouacer, Veronique Deiler, Myriam Moussa, Atika Mouaci Nassima Viguier, Sophie Letrou

**Affiliations:** 1 UMR 1137, IAME, Université Paris Diderot, F75018 Paris, France; 2 Medical and Infectious Diseases Care Unit, AP-HP, Bichat University Hospital, F75018 Paris, France; 3 Service de Réanimation Médicale et des Maladies Infectieuses, Hôpital Bichat–Claude-Bernard, 46 rue Henri-Huchard, 75877 Paris Cedex 18, France; 4 AP-HP, Pitié-Salpêtrière University Hospital, Cardiology, Paris, France; 5 APHP, Saint-Louis University Hospital, Medical Intensive Care Unit, Paris, France; 6 Paris-7 Paris Diderot University, Paris, France; 7 Medical ICU, Edouard Herriot University Hospital, Lyon, France; 8 Critical Care Medicine Unit CH Etampes-Dourdan, Etampes, France; 9 Medical Unit, French British Hospital Institute, Levallois-Perret, France; 10 Respiratory and Infectious Diseases ICU, APHM Hôpital Nord, Aix Marseille University, Marseille, France; 11 AP-HP, Avicenne Hospital, Intensive Care Unit, Paris, France; 12 Medicine University, Paris 13 University, Bobigny, France; 13 Surgical Intensive Care Unit and Lyon University Hospital, Lyon, France; 14 OUTCOMEREA organization, F75018 Paris, France; 15 Medical Intensive Care Unit and University Hospital Centre, Nantes, France; 16 Medical Intensive Care Unit, Gabriel Montpied University Hospital, Clermont-Ferrand, France; 17 Medical Intensive Care Unit, Grenoble University Hospital, Grenoble 1 University, U823 La Tronche, France; 18 Outcomerea research network, 93000 Aulnay-sous-Bois, France

**Keywords:** Potassium, Correction of potassium, Critical care, Mortality, Cardiac events

## Abstract

**Objectives:**

Our objectives were (1) to characterize the distribution of serum potassium levels at ICU admission, (2) to examine the relationship between dyskalemia at ICU admission and occurrence of cardiac events, and (3) to study both the association between dyskalemia at ICU admission and dyskalemia correction by day 2 on 28-day mortality.

**Design:**

Inception cohort study from the longitudinal prospective French multicenter OUTCOMEREA database (1999–2014)

**Setting:**

22 French OUTCOMEREA network ICUs

**Patients:**

Patients were classified into six groups according to their serum potassium level at admission: three groups of hypokalemia and three groups of hyperkalemia defined as serious hypokalemia [K+] < 2.5 and serious hyperkalemia [K+] > 7 mmol/L, moderate hypokalemia 2.5 ≤ [K+] < 3 mmol/L and moderate hyperkalemia 6 < [K+] ≤ 7 mmol/L, and mild hypokalemia 3 ≤ [K+] < 3.5 mmol/L and mild hyperkalemia 5 < [K+] ≤ 6 mmol/L. We sorted evolution at day 2 of dyskalemia into three categories: balanced, not-balanced, and overbalanced.

**Intervention:**

None

**Measurements and main results:**

Of 12,090 patients, 2108 (17.4%) had hypokalemia and 1445 (12%) had hyperkalemia.

Prognostic impact of dyskalemia and its correction was assessed using multivariate Cox models. After adjustment, hypokalemia and hyperkalemia were independently associated with a greater risk of 28-day mortality. Mild hyperkalemic patients had the highest mortality (hazard ratio (HR) 1.29, 95% confidence interval (CI) [1.13–1.47], *p* < 0.001). Adjusted 28-day mortality was higher if serum potassium level was not-balanced at day 2 (aHR = 1.51, 95% CI [1.30–1.76], *p* < 0.0001) and numerically higher but not significantly different if serum potassium level was overbalanced at day 2 (aHR = 1.157, 95% CI [0.84–1.60], *p* = 0.38). Occurrence of cardiac events was evaluated by logistic regression. Except for patients with serious hypokalemia at admission, the depth of dyskalemia was associated with increased risk of cardiac events.

**Conclusions:**

Dyskalemia is common at ICU admission and associated with increased mortality. Occurrence of cardiac events increased with dyskalemia depth. A correction of serum potassium level by day 2 was associated with improved prognosis.

## Introduction

The relationship between imbalances in potassium homeostasis and cardiac events has been well established for many years [1]. Current guidelines recommend that in patients with acute myocardial infarction serum potassium levels be maintained from 4.0 to 5.0 mmol/L or even 4.5 to 5.5 mmol/L [2]. Moreover, dyskalemia defined as a serum potassium level [K^+^] < 3 mmol/L or [K^+^] ≥ 5 mmol/L has been incorporated into the Simplified Acute Physiology Score (SAPS II) a risk prediction model in intensive care unit (ICU) [3]. In a recent and large two-center retrospective study [4], high serum potassium level at critical care initiation was associated with an increased risk of death even at moderate increase above normal. In the same study, a decrease in serum potassium concentration ≥ 1 mmol/L within 48 h in patients with hyperkalemia after ICU admission made hyperkalemia and adjusted risk of mortality no longer significant. However, this study was retrospective and bi-centric and it cannot be ruled out that the observed results on mortality may have been explained by other factors not recorded. Moreover, this study did not evaluate the full range of serum potassium level at admission. Therefore, considering the lack of data on outcomes of dyskalemia and persistent dyskalemia in ICU patients, it is critical to extend our knowledge on the subject.

Our objectives were (1) to characterize the distribution of serum potassium levels at ICU admission, (2) to examine the relationship between dyskalemia at ICU admission and occurrence of cardiac events, and (3) to study both the association between dyskalemia at ICU admission and dyskalemia correction by day 2 on 28-day mortality.

## Methods

### Study design and data source

We conducted an observational study on a prospective multicenter database (OutcomeRea; www.outcomerea.org) fed by 22 French ICUs between April 1999 and January 2014. Details about the database and its quality have already been described [5–8].

According to the French law, the OUTCOMEREA database was declared to the Commission Nationale de l’Informatique et des Libertés. This study did not require individual patient consent, because it involved research on a previously developed and approved database. The institutional review board of the Rhône-Alpes-Auvergne Clinical Investigation Centers approved this study.

### Study population, data collection, and definitions

We included consecutive patients who were older than 18 years at ICU admission and alive on day 2. We excluded patients without serum potassium level measure at admission, patients with less than 2 serum potassium level measures during ICU stay, and patients with at least 2 consecutive days without serum potassium measures.

Only the first ICU admission was considered. The following information was recorded: age, sex, admission category, origin, SAPS II [3], and Sequential Organ Failure Assessment (SOFA) score [9], and Knaus Scale definitions were used to record pre-existing chronic organ failures, including respiratory, cardiac, hepatic, renal, and immunological dysfunctions [10], and cardiac events.

In the absence of recommendations for ICU patients, normal kalemia was defined as a serum potassium level between 3 and 5 mmol/L in agreement with the SAPS II. However, we delineated two more groups as borderline dyskalemia. Overall, we studied six groups of dyskalemia, three groups of hypokalemia and three groups of hyperkalemia defined as serious hypokalemia [K+] < 2.5 and serious hyperkalemia [K+] > 7 mmol/L, moderate hypokalemia 2.5 ≤ [K+] < 3 mmol/L and moderate hyperkalemia 6 < [K+] ≤ 7 mmol/L, and mild hypokalemia 3 ≤ [K+] < 3.5 mmol/L and mild hyperkalemia 5 < [K+] ≤ 6 mmol/L.

We sorted evolution of serum potassium level on day 2 into three categories: “balanced kalemia” = serum potassium level normal on day 2, “not-balanced kalemia” = serum potassium level still not corrected, and “overbalanced kalemia” = serum potassium level reversed from hypo- to hyperkalemia or the opposite.

The missing values of serum potassium level at D2 (*N* = 114) were substituted by the mean of serum potassium levels measured on day 1 and day 3.

### Statistical analysis

The primary outcome was the 28-day mortality following admission in ICU. Secondary outcomes were cardiac events within the first 48 h of ICU stay prospectively recorded into the OUTCOMEREA database: external electric shock, cardiac arrest, myocardial infarction, minimal heart rate < 40 beats per minute, ventricular arrhythmia, supra-ventricular arrhythmia, unspecified arrhythmia, and atrioventricular block. To summarize the data, we create a variable named “cardiac events” rated 1 if at least one of the cardiac events cited above occurred within the first 48 h.

Categorical variables are described as number (%) and continuous variables as median (interquartile range, IQR). For comparisons, we used the chi-square test for categorical data and the Mann-Whitney *U* test for continuous data.

Univariate Cox models, stratified on ICU centers, were developed to assess the independent effects on 28-day mortality. Factors included in the initial models were sex, age older than 60 years, medical admission, maximum SOFA at day 1 or 2 without renal score, main symptom at admission, underlying cardiovascular and renal conditions and immunodeficiency, variables found to be significant to the *p* < 0.10 level in univariate analyses. Stepwise variable elimination was then performed on the multivariate model to develop the most parsimonious model. Two-by-two clinically sound interactions were tested. In order to mitigate the influence of less predictable prognosis associated with asymptomatic hyperkalemia related to chronic renal failure, we forced chronic renal disease and renal replacement therapy at day 1 (D1) or D2 in the multivariate model for prognosis assessment of admission serum potassium level.

Effect of serum potassium level at admission on the 28-day mortality was assessed with a multivariate Cox model, stratified on ICU centers and adjusted on the previously identified factors independently associated with 28-day mortality. The proportionality of hazard was assessed using graphical methods that allowed concluding that the proportionality was respected.

The occurrence of cardiac events was evaluated by logistic regression stratified on ICU centers and adjusted on factors independently associated with the 28-day mortality.

Last, sensitivity analyses on patients with each category of hypokalemia or hyperkalemia at admission were performed to assess the influence of the serum potassium evolution (not-balanced, balanced, or overbalanced) on the 28-day mortality. A multivariate Cox model, stratified on the ICU center, adjusted on the independent factors of 28-day mortality, and adjusted on serum potassium level at admission, was used.

Values of *p* < 0.05 were considered significant. Analyses were performed using SAS 9.4 software (SAS Institute, Cary, NC).

## Results

### Study population

**Fig. 1:**
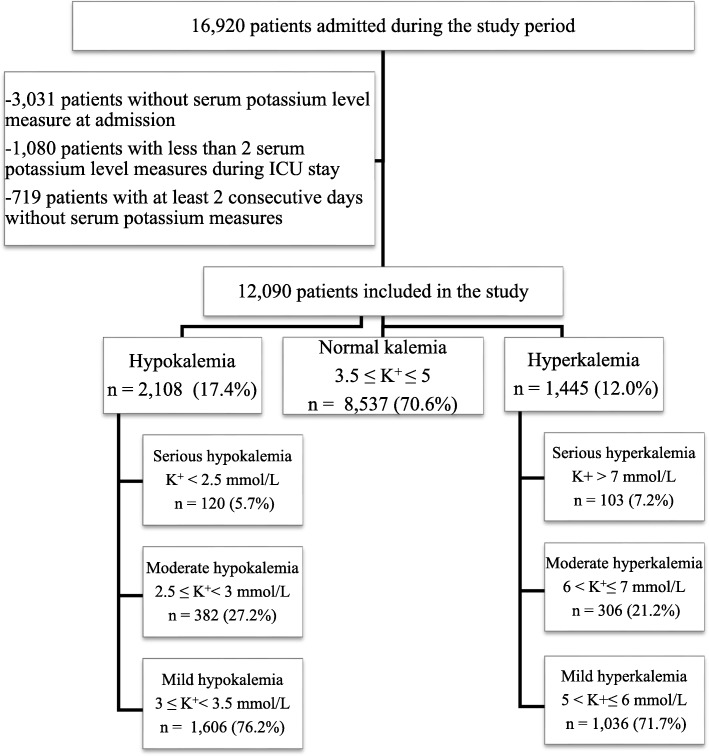
Flow chart

Of the 16,920 patients entered into the database during the study period, 12,090 fulfilled our inclusion criteria. Overall, the prevalence of dyskalemia at admission was 29.4%: 2108 patients (17.4%) had serious to mild hypokalemia, 8537 (70.6%) patients had no dyskalemia, and 1445 (12.0%) had mild to serious hyperkalemia. The flow chart is provided in Fig. 1. The distribution of admission serum potassium levels approximated that of a normal distribution (Additional file 1: Figure S1), with a median of serum potassium level at admission of 4.0[3.6; 4.6] mmol/L.

### Characteristics of patients at admission

Baseline characteristics of the 12,090 study patients by groups of serum potassium level at admission are shown in Additional file 1: Table S1. The relationship between potassium levels and baseline variables was complex. The relationship was U-shaped for SAPS II, SOFA scores, and medical admission, while it was without any specific shape for other variables. Medical patients were more likely to have dyskalemia (31%, 2845/9091) than surgical patients (23%, 678/2999, *p* < 0.01) (Additional file 1: Figure S2). The prevalence of dyskalemia at ICU admission varied according to comorbidities (Additional file 1: Figure S3 and S4). The highest prevalence of hyperkalemia and hypokalemia was observed in patients with a chronic renal disease (27%) and in immunosuppressed patients (21%), respectively.

### Impact of dyskalemia at admission on cardiac events and 28-day mortality

**Fig. 2:**
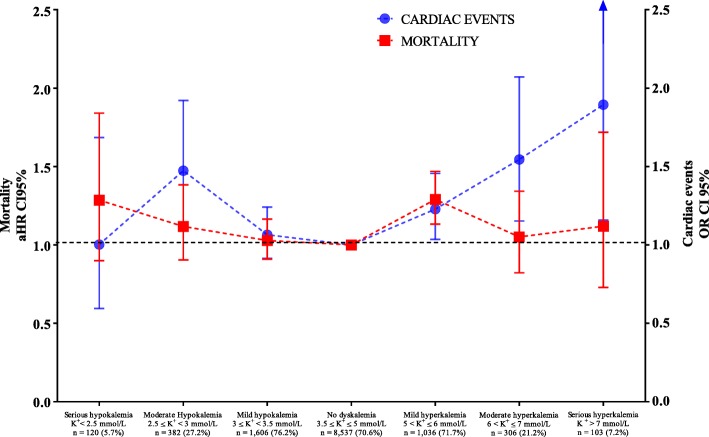
28-day mortality and cardiac events within the first 48 h in ICU by the potassium blood level at admission. Abbreviations: aHR adjusted hazard ratio, OR odds ratio. Adjusted on age above 60 years, chronic illness (cardiac, renal and immune-depression), main cause of admission, SOFA without renal score and medical admission. Numbers of cardiac and subsequent rates for each group are listed in Table 2

Of the 12,090 study patients, 2269 (18.8%, 95% confidence interval (CI) 18.1–19.5%) died before day 28. Details by group are provided in Fig. 1. After adjustment for confounders, hypokalemia and hyperkalemia were independently associated with a greater risk of 28-day adjusted mortality. There was a U-shaped relationship between the level of dyskalemia at admission and the adjusted mortality (Fig. 2). This U-shaped relationship was only slightly tempered because the highest risk of mortality was observed in mild hyperkalemic patients (hazard ratio (HR) 1.29, 95% CI [1.13–1.47], *p* < 0.001) (Fig. 2). All independent factors associated with increased 28-day mortality are listed in Table 1. Except for patients with serious hypokalemia at admission, the deeper the dyskalemia is at ICU admission, the more is their risk of cardiac events. Cardiac event types by each group of patients are provided in Table 2. The risk of cardiac events did not pair entirely with the mortality risk (Fig. 2). All independent factors associated with the occurrence of cardiac events within the first 48 h are listed in Table 3.

**Table 1:** Factors independently associated with 28-day mortality

	aHR	95% CI	*p* value
Potassium level at admission			
Serious hypokalemia (K^+^ < 2.5 mmol/L)	1.286	0.899–1.840	0.17
Moderate hypokalemia (2.5 ≤ K^+^ < 3 mmol/L)	1.118	0.904–1.384	0.30
Mild hypokalemia (3 ≤ K^+^ < 3.5 mmol/L)	1.028	0.909–1.164	0.66
No dyskalemia (3.5 ≤ K^+^ ≤ 5 mmol/L)	1	Ref	0.01
Mild hyperkalemia (5 < K^+^ ≤ 6 mmol/L)	1.290	1.133–1.469	0.01
Moderate hyperkalemia (6 < K^+^ ≤ 7 mmol/L)	1.051	0.822–1.343	0.69
Serious hyperkalemia (K^+^ > 7 mmol/L)	1.119	0.729–1.719	0.61
Renal replacement therapy on day 1 or on day 2	1.337	1.184–1.510	< 0.01
Age > 60 years	1.867	1.695–2.057	< 0.01
Underlying disease			
Cardiac	1.355	1.211–1.516	< 0.01
Immunosuppression	1.366	1.221–1.528	< 0.01
Admission category, medical	1.304	1.156–1.471	< 0.01
Main symptom admission			
Shock and multiple organ failure	1.925	1.635–2.267	< 0.01
Acute respiratory failure and COPD exacerbation	1.646	1.392–1.945	< 0.01
Acute renal failure	1.238	0.947–1.618	0.12
Coma	2.337	1.960–2.787	< 0.01
Trauma, monitoring, scheduled surgery	1	Ref	< 0.01
SOFA score without points for renal failure	1.214	1.198–1.230	< 0.01

*Abbreviations*: *COPD* chronic obstructive pulmonary disease, *SOFA* Sequential Organ Failure Assessment

Adjusted hazard ratio (aHR) on age above 60 years, chronic illness (cardiac, renal and immune-depression), main cause of admission, SOFA without renal score and medical critical disease and 95% confidence intervals (95% CI)

**Table 2:** Cardiac events within the first 48 h by potassium level at admission

Cardiac events	Serious hypokalemia K^+^ < 2.5 mmol/L *N* = 120	Moderate hypokalemia 2.5 ≤ K^+^ < 3 mmol/L *N* = 382	Mild hypokalemia 3 ≤ K^+^ < 3.5 mmol/L *N* = 1606	No dyskalemia 3.5 ≤ K^+^ ≤ 5 mmol/L *N* = 8537	Mild hyperkalemia 5 < K^+^ ≤ 6 mmol/L *N* = 1036	Moderate hyperkalemia 6 < K^+^ ≤ 7 mmol/L *N* = 306	Serious hyperkalemia K^+^ > 7 mmol/L *N* = 103	*p* value*
External electric shock	0 (0)	6 (1.6)	8 (0.5)	71 (0.8)	13 (1.3)	0 (0)	3 (2.9)	0.01
Cardiac arrest	1 (0.8)	14 (3.7)	23 (1.4)	132 (1.5)	31 (3)	13 (4.2)	6 (5.8)	< 0.01
Myocardial infarction	1 (0.8)	1 (0.3)	4 (0.2)	21 (0.2)	5 (0.5)	2 (0.7)	0 (0)	0.54
Minimal heart rate < 40 bpm	10 (8.3)	27 (7.1)	79 (4.9)	333 (3.9)	79 (7.6)	40 (13.1)	17 (16.5)	< 0.01
Ventricular arrhythmia	10 (8.3)	43 (11.3)	103 (6.4)	420 (4.9)	73 (7)	31 (10.1)	14 (13.6)	< 0.01
Supra-ventricular arrhythmia	6 (5)	30 (7.9)	105 (6.5)	680 (8)	109 (10.5)	31 (10.1)	7 (6.8)	< 0.01
Unspecified arrhythmia	0 (0)	1 (0.3)	8 (0.5)	35 (0.4)	4 (0.4)	1 (0.3)	0 (0)	0.96
Atrioventricular block	0 (0)	0 (0)	2 (0.1)	1 (0)	0 (0)	0 (0)	0 (0)	0.28
Total	20 (16.7)	87 (22.8)	267 (16.6)	1327 (15.5)	239 (23.1)	83 (27.1)	30 (29.1)	< 0.01

Cardiac event was achieved if a patient experiment at least one cardiac event in the first 2 days. Results are expressed in number of patients (*n*) and percentage (%)

*Comparison across serum potassium concentration categories at ICU admission

**Table 3:** Factors independently associated with occurrence of cardiac events

	aOR	95% CI	*p* value
Potassium level at admission			
Serious hypokalemia (K^+^ < 2.5 mmol/L)	1.002	0.595–1.585	1.00
Moderate hypokalemia (2.5 ≤ K^+^ < 3 mmol/L)	1.473	1.130–1.921	< 0.01
Mild hypokalemia (3 ≤ K^+^ < 3.5 mmol/L)	1.065	0.914–1.242	0.42
No dyskalemia (3.5 ≤ K^+^ ≤ 5 mmol/L)	1	Reference	< 0.01*
Mild hyperkalemia (5 < K^+^ ≤ 6 mmol/L)	1.228	1.035–1.456	0.02
Moderate hyperkalemia (6 < K^+^ ≤ 7 mmol/L)	1.495	1.153–2.071	< 0.01
Serious hyperkalemia (K^+^ > 7 mmol/L)	1.894	1.158–3.100	0.01
Renal replacement therapy day 1 or day 2	1.126	0.914–1.388	< 0.01
Age > 60 years	2.316	2.062–2.602	< 0.01
Underlying disease			
Cardiac	1.809	1.579–2.073	< 0.01
Immunosuppression	0.761	0.651–0.890	< 0.01
Admission category, medical	1.442	1.244–1.671	< 0.01
Main symptom admission			
Shock and multiple organ failure	1.428	1.210–1.685	< 0.01
Acute respiratory failure and COPD exacerbation	1.133	0.956–1.342	0.15
Acute renal failure	0.752	0.521–0.946	0.02
Coma	1.559	1.298–1.873	< 0.01
Trauma, monitoring, scheduled surgery	1	Ref	< 0.01
SOFA score without points for renal failure	1.185	1.166–1.205	< 0.01

*Abbreviations*: *COPD* chronic obstructive pulmonary disease, *SOFA* Sequential Organ Failure Assessment

Adjusted odds ratio (aOR) on age above 60 years, chronic illness (cardiac, renal and immune-depression), main cause of admission, SOFA without renal score and medical critical disease, and 95% confidence intervals (95% CI)

*The *P* value indicates that there are significant differences overall between level of kalemia

### Impact on 28-day mortality of persistent (not-balanced or overbalanced) dyskalemia within the first 2 days in ICU

Correction of dyskalemia at admission was achieved within the two first days in ICU in 1067 (50.6%) hypokalemic and 908 (62.8%) hyperkalemic patients at admission (Additional file 1: Table S2). Dyskalemia was not-balanced in 1005 (47.7%) and 427 (29.5%) and overbalanced in 36 (1.7%) and in 110 (7.6%) hypokalemic and hyperkalemic patients at admission, respectively (Additional file 1: Table S2).

**Fig. 3:**
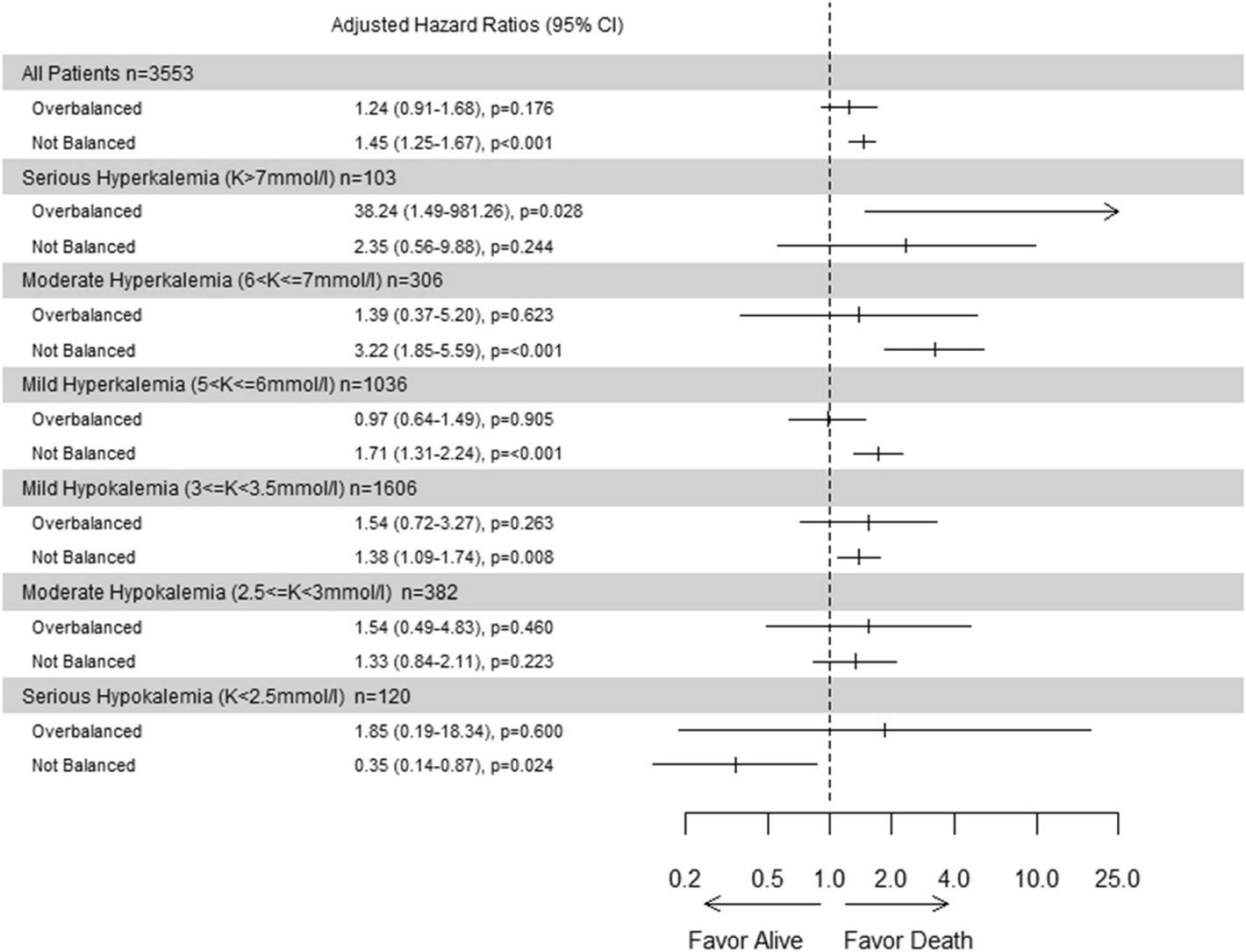
28-day mortality by persistent dyskalemia at day 2

After adjustment on risk factors of death at day 28, risk of dying was significantly higher if serum potassium level was not-balanced (aHR = 1.45, 95% CI [1.25; 1.67], *p* < 0.001) and numerically higher but not significantly different if serum potassium level was overbalanced (aHR = 1.24, 95% CI [0.91; 1.68], *p* = 0.176). Details for each group of patients with dyskalemia at admission are shown in Fig. 3.

## Discussion

In this large multicenter cohort of unselected ICU patients, we found a high prevalence of dyskalemia at ICU admission. Dyskalemia was independently associated with a greater risk of 28-day mortality after careful adjustment on confounders. The depth of dyskalemia was closely associated with increased risk of cardiac events. However, the risk of cardiac events did not pair entirely with the mortality risk. Moreover, in patients with abnormal serum potassium level on admission, the correction of dyskalemia at day 2 was independently associated with 28-day survival.

Hessels et al. [11] found an incidence of hypokalemia of 22% and an incidence of hyperkalemia of 20.1% while McMahon et al. [4] found an incidence of hyperkalemia of 22%. Compare to the two latter studies in ICU, incidences of dyskalemia were lower in our cohort. Discrepancies might be due to the fact that the authors retained the highest value of serum potassium level measured on the day of critical care initiation [4] or considered all the values within the first day in ICU and patients were both classified as hypokalemic or hyperkalemic patients’ category [11]. The differences in patients’ case-mix from the two studies may also have played a role to explain the discrepancies between studies.

The prevalence of dyskalemia at ICU admission varied according to comorbidities: higher prevalence of hyperkalemia and hypokalemia were found in patients with a chronic renal disease (27%) and in immunosuppressed patients (21%) respectively. The burden of chronic kidney disease is rising [12], a well-known comorbidity associated with kalemia disturbance [13]. Henz et al. [14] quantified the impact of drugs and comorbidity on serum potassium level in 15,000 unselected patients admitted to the hospital. They identified the renal function as the strongest single predictor of serum potassium level at hospital admission. Frequent hypokalemia in immunocompromised patients could be due to corticosteroid intake, which is a common adjuvant therapy in those patients, or related to tubulopathy, a frequent adverse event of chemotherapy [15].

Increased mortality associated with potassium disturbances at admission in different settings has been described previously [4, 11]. In keeping with previous findings, we found that dyskalemia was independently associated with a higher risk of 28-day mortality after careful adjustment on confounding factors. McMahon et al. [4] found that hyperkalemia was a strong predictor of all-cause mortality within 30 days following critical care initiation, with a significant risk gradient across potassium groups following multivariable adjustment. This U-shaped relationship between potassium levels and mortality was confirmed by Hessels et al. for both hypokalemia and hyperkalemia [11].

The mechanism for increased 28-day mortality following ICU admission in patients with dyskalemia may be related to life-threatening cardiac events. However, the degree of potassium disturbance alone is usually considered as insufficient to explain life-threatening cardiac events at least for non-severe disturbances. Indeed, in patients after acute myocardial infarction, Goyal et al. [2] showed that the relationship between potassium levels and the rate of ventricular fibrillation or cardiac arrest was relatively flat across a wide range of post-admission potassium mean levels, except for extreme values (3.0 and 5.0 mmol/L). In contrast to Goyal et al.’s study, we found that the depth of dyskalemia was closely associated with an increased risk of cardiac events. This discrepancy between our results and the data from Goyal et al. may be related to our population of patients, more susceptible to the degree of potassium disturbance because of their underlying comorbidities or their severity of illness.

The risk of cardiac events did not pair with the mortality risk. Indeed, the mild hyperkalemic group had a higher risk of mortality but not a higher risk of cardiac event occurrence. We hypothesized that in this group many patients had a mild hyperkalemia related to respiratory acidosis, as this group of patients had more chronic respiratory disease, were more frequently admitted for an acute respiratory failure or a COPD exacerbation, had a greater respiratory SOFA score, and were more frequently under MV or NVI (Additional file 1: Figure S5). The cardiac events experienced by patients in this group could have been less easy to resuscitate and, although less numerous, more often fatal. Indeed, myocardial acidosis associated with hypercarbonic acidosis reduces the rate of successful cardiac resuscitation [16, 17].

Although the first 48 h of cardiac events in ICU seems a relatively short time interval to explain the excess of mortality at day 28, the negative hemodynamic consequences of such cardiac events in critically ill unstable patients might not disappear immediately with the resolution of the event. In addition, cardiac events imply drug treatment such as antiarrhythmic or trigger the initiation of therapeutic anticoagulation and subsequently an increased risk of side effects. Together, these effects could be catastrophic in severely ill patients. Recently, Klein Klouwenberg et al. showed that very short episodes of atrial fibrillation were independently associated with excess mortality in a large cohort of critically ill patients with sepsis [18].

For dyskalemic patients at admission, we observed that an unbalanced dyskalemia after 2 days in ICU was significantly associated with 28-day mortality. This finding is supported by the recent study from Hessels et al. which demonstrated that in ICU patients, potassium variability (defined as the standard deviation of all potassium levels both on day 1 (early phase) and from day 2 to day 7 (late phase)) was independently associated with increased mortality. Similarly, McMahon et al. [4] found that in hyperkalemic patients at ICU admission who had a decrease ≥ 1 mmol/L in 48 h post-critical care initiation, the association between high serum potassium levels at ICU admission and mortality is no longer significant. However, the methods of the two latter studies have significant weaknesses. Hessels et al. [11] focused their study on variability, a parameter not easy to apprehend in real time for a physician. McMahon et al. [4] studied only hyperkalemic patients and did not take into account any overbalanced correction of the initial hyperkalemia. Our results bring knowledge on the topic in comparison to the two previous studies. First, we found that after adjustment, both hypokalemia and hyperkalemia were independently associated with a greater risk of 28-day mortality. Second, we considered not-balanced and overbalanced corrections, much more relevant parameters at the bedside than variability. As this study was observational, we cannot exclude that dyskalemia correction was a marker of a change in the same way in many other parameters, for instance pH has an important effect on serum potassium levels, instead of reflecting a direct causal impact of serum potassium level.

The strengths of our study include the use of a national multicenter database, a large study size with 12,090 critically ill patients, the prospective data collection of cardiac events, and the use of a model to minimize the effects of confounding factors such as baseline imbalances.

Our study also has several limitations. First, there is a potential for biases due to self-selection of ICUs joining the OUTCOMEREA database. Second, although it is multicenter, our database is not multinational. Third, many important data are lacking in our database to interpret serum potassium levels, example being degrees of chronic disease or nutritional status of the patients. Moreover, the missing values of serum potassium level at day 2 were substituted by the mean potassium levels on day 1 and day3. Fourth, we cannot rule out some misclassifications of not-balanced and overbalanced patients as potassium measurements were performed at the physician’s discretion. Fifth, because of the observational type of the study, there is always a risk that observational data cannot elicit complex etiologic relations. For instance, many factors may have influenced cardiac events and were not assessed such as catecholamine therapy or on the contrary antiarrhythmic agents. Finally, all the care protocols and the management of dyskalemia likely varied between institutions. Thus, it remains unknown whether dyskalemia is a true cause of excess mortality and amenable to treatment or merely a marker of ICU patients complexity. In conclusion, relations between potassium management and outcome can only be investigated in a large randomized study [19–21].

## Conclusion

Dyskalemia is common at ICU admission and associated with increased mortality. Occurrence of cardiac events increased with dyskalemia depth. A correction of serum potassium level by day 2 improved prognosis.

## Supplementary information

**Additional file 1: Table S1.** Baseline characteristics by serum potassium level at admission. **Table S2.** Crude mortality according to the dyskalemia at ICU admission and its type of correction (balanced, not-balanced, or overbalanced). **Figure S1.** Distribution of serum potassium levels at admission in the overall population. **Figure S2.** Prevalence of dyskalemia in medical and surgical patients. **Figure S3.** Prevalence of hypokalemia according to the presence of underlying disease. **Figure S4.** Prevalence of hyperkalemia according to the presence of underlying disease. **Figure S5.** Characteristics of mild hyperkalemic patients at admission.

## Data Availability

All the data and material are available from ^1^Outcomerea research network; Aulnay Sous Bois, 93000 France, http://outcomerea.fr/index.php/contact.
