# Supplementary material for: Influence of dyskalemia at admission and early dyskalemia correction on survival and cardiac events of critically ill patients
Source: Crit Care. 2019 Dec 19;23:415. doi: 10.1186/s13054-019-2679-z (PMC6921444; doi:10.1186/s13054-019-2679-z)
Supplement: Supplementary file 1 — Additional file 1: Table S1. Baseline characteristics by serum potassium level at admission. Table S2. Crude mortality according to the dyskalemia at ICU admission and its type of correction (balanced, not-balanced, or overbalanced). Figure S1. Distribution of serum potassium levels at admission in the overall population. Figure S2. Prevalence of dyskalemia in medical and surgical patients. Figure S3. Prevalence of hypokalemia according to the presence of underlying disease. Figure S4. Prevalence of hyperkalemia according to the presence of underlying disease. Figure S5. Characteristics of mild hyperkalemic patients at admission. [file 13054_2019_2679_MOESM1_ESM.docx]

## Additional file 1

| Additional Table 1. Baseline characteristics by serum potassium level at admission | | | | | | | | | |
| --- | --- | --- | --- | --- | --- | --- | --- | --- | --- |
| Baseline characteristics | The whole population of patients  N = 12090 | Serious  hypokalemia  K^+^ < 2.5 mmol/L  N = 120 | Moderate  hypokalemia  2.5 ≤ K^+^< 3 mmol/L  N = 382 | Mild  hypokalemia  3 ≤ K^+^< 3.5 mmol/L  N = 1606 | No  dyskalemia  3.5 ≤ K^+^ ≤ 5 mmol/L  N = 8537 | Mild  hyperkalemia  5 < K^+^ ≤ 6 mmol/L  N = 1036 | Moderate  hyperkalemia  6 < K^+^ ≤ 7 mmol/L  N = 306 | Serious  hyperkalemia  K^+^ > 7 mmol/L  N = 103 | p-value* |
| **Patient characteristics** |  |  |  |  |  |  |  |  |  |
| Male gender | 7318 (60.5) | 49 (40.8) | 188 (49.2) | 804 (50.1) | 5349 (62.7) | 660 (63.7) | 202 (66) | 66 (64.1) | <.01 |
| Age (years), | 63 [49.2;75.7] | 56.6 [44.3;71.2] | 57.9 [46.4;70.7] | 60.4 [46.2;73.7] | 62.9 [49;75.5] | 69.1 [56.5;78.3] | 65.9 [53.3;76.4] | 68 [51.4;78.5] | <.01 |
| **Underlying disease** |  |  |  |  |  |  |  |  |  |
| Hepatic | 758 (6.3) | 7 (5.8) | 37 (9.7) | 78 (4.9) | 497 (5.8) | 96 (9.3) | 36 (11.8) | 7 (6.8) | <.01 |
| Cardiac | 1711 (14.2) | 7 (5.8) | 41 (10.7) | 175 (10.9) | 1179 (13.8) | 219 (21.1) | 75 (24.5) | 15 (14.6) | <.01 |
| Pulmonary | 1739 (14.4) | 4 (3.3) | 28 (7.3) | 185 (11.5) | 1284 (15) | 188 (18.1) | 40 (13.1) | 10 (9.7) | <.01 |
| Renal | 772 (6.4) | 6 (5) | 16 (4.2) | 64 (4) | 474 (5.6) | 131 (12.6) | 64 (20.9) | 17 (16.5) | <.01 |
| Immunosuppression | 1989 (16.5) | 25 (20.8) | 94 (24.6) | 302 (18.8) | 1347 (15.8) | 171 (16.5) | 38 (12.4) | 12 (11.7) | <.01 |
| **Admission category, medical** | 9091 (75.2) | 106 (88.3) | 329 (86.1) | 1307 (81.4) | 6246 (73.2) | 767 (74) | 245 (80.1) | 91 (88.3) | <.01 |
| **Main symptom admission** |  |  |  |  |  |  |  |  | <.01 |
| Shock and multiple organ failure | 2814 (23.3) | 37 (30.8) | 104 (27.2) | 391 (24.3) | 1828 (21.4) | 337 (32.5) | 87 (28.4) | 30 (29.1) |  |
| ARF failure and COPD exacerbation | 3265 (27.2) | 21 (17.5) | 86 (22.5) | 418 (26) | 2408 (28.2) | 271 (26.2) | 52 (17) | 9 (8.7) |  |
| Acute renal failure | 644 (5.4) | 5 (4.2) | 16 (4.2) | 52 (3.2) | 329 (3.9) | 113 (10.9) | 92 (30.1) | 37 (35.9) |  |
| Coma | 1992 (16.6) | 31 (25.8) | 98 (25.7) | 346 (21.5) | 1398 (16.4) | 83 (8) | 27 (8.8) | 9 (8.7) |  |
| Trauma, monitoring, scheduled surgery | 3309 (27.5) | 26(21.7) | 78 (20.4) | 399 (24.8) | 2574 (30.2 | 232 (22.4) | 48 (15.7) | 18 (17.5) |  |
| **Scores** |  |  |  |  |  |  |  |  |  |
| SAPS II, points (3) | 41 [30 ; 53] | 45.5 [34;58] | 44 [34;58] | 41[30;53] | 39 [29;51] | 49 [38.5;62] | 50 [41;62] | 52 [39;68] | <.01 |
| SOFA, points (10) | 5 [3 ; 8] | 6 [4;9] | 7 [4;10] | 5 [3;8] | 5 [3;8] | 7 [4;10] | 7 [5;10] | 7 [5;11] | <.01 |
| **Procedures within 48 hours** |  |  |  |  |  |  |  |  |  |
| MV/NIV | 7610 (62.9) | 60 (50) | 237 (62) | 1001 (62.3) | 5407 (63.3) | 687 (66.3) | 166 (54.2) | 52 (50.5) | <.01 |
| HD | 1207 (10) | 8 (6.7) | 27 (7.1) | 106 (6.6) | 632 (7.4) | 242 (23.4) | 132 (43.1) | 60 (58.3) | <.01 |
| **Outcomes** |  |  |  |  |  |  |  |  |  |
| **ICU length of stay (days)** | 5 [3 ; 9] | 4 [3;11] | 5 [3;10] | 5[3;9] | 5 [3;9] | 5 [3;11] | 4 [3;10] | 4 [2;7] | 0.03 |
| **Hospital length of stay (days)** | 17 [9 ; 32] | 16 [9;32] | 18 [8;33] | 17 [9;33] | 17 [9;32] | 18 [9;31] | 17 [7;36] | 15 [6;31] | 0.77 |
| **Crude ICU mortality (days)** | 1883 (15.6) | 26 (21.7) | 81 (21.2) | 254 [15.8] | 1207 (24.1) | 240 (23.2) | 59 (19.3) | 16 (15.5) | <.01 |
| **Crude 28-day mortality** | 2269 (18.8) | 31 (25.8) | 92 (24.1) | 309 (19.2) | 1460 (17.1) | 285 (27.5) | 70 (22.9) | 22 (21.4) | <.01 |
| Results are presented as number (percentage), mean (standard deviation and median [interquartile range] for qualitative and quantitative variables accordingly.  Abbreviations: ARF = Acute respiratory failure; COPD = chronic obstructive pulmonary disease; SAPS II = Simplified Acute Physiology Score II; SOFA = Sequential Organ failure Assessment; MV = Mechanical ventilation; NVI = Non Invasive Ventilation; HD = hemodialysis; ICU = Intensive Care Unit  * Comparison across serum potassium sodium concentration categories at ICU admission | | | | | | | | | |

| Additional Table 2. Crude mortality according to the dyskalemia status at ICU admission and its type of correction (balanced, not-balanced, or overbalanced) | | | | | | |
| --- | --- | --- | --- | --- | --- | --- |
| DYSKALEMIA AT ADMISSION | NOT-BALANCED | | BALANCED | | OVER BALANCED | |
|  | n (%) | Mortality  n, (%) | n (%) | Mortality  n, (%) | n (%) | Mortality,  n, (%) |
| Serious hypokalemia  K^+^ < 2.5 mmol/L  N = 120 | 61 (50.8) | 14 (23.0) | 57 (47.5) | 16 (28.1) | 2 (1.7) | 1 (50.0) |
| Moderate hypokalemia  2.5 ≤ K^+^< 3mmol/L  N = 382 | 208 (54.5) | 57 (27.4) | 167 (43.7) | 31 (18.6) | 7 (1.8) | 4 (57.1) |
| Mild hypokalemia  3 ≤ K^+^< 3.5 mmol/L  N = 1606 | 736 (45.8) | 157 (21.3) | 843 (52.5) | 144 (17.1) | 27 (1.7) | 8 (29.6) |
| Overall, hypokalemic patients  N = 2108 | 1005 (47.7) | 228 (10.8) | 1067 (50.6) | 191( 9.1) | 36 (1.7) | 13 (0.6) |
| Mild hyperkalemia  5 < K^+^ ≤ 6 mmol/L  N = 1036 | 280 (27.0) | 92 (32.9) | 678 (65.4) | 166 (24.5) | 78 (7.5) | 27 (24.6) |
| Moderate hyperkalemia  6 < K^+^ ≤ 7 mmol/L  N = 306 | 106 (34.6) | 40 (37.7) | 178 (58.2) | 26 (14.6) | 22 (7.2) | 4 (18.2) |
| Serious hyperkalemia  K^+^ > 7 mmol/L  N = 103 | 41 (39.8) | 14 (34.2) | 52 (50.5) | 6 (11.5) | 10 (9.7) | 2 (20) |
| Overall, hyperkalemic patients  N = 1445 | 427 (29.5) | 146 (10.1) | 908 (62.8) | 198 (3.7) | 110 (7.6) | 33 (2.3) |
| Balanced kalemia = kalemia was normal at day 2; Not-balanced kalemia = kalemia remained not corrected at day 2 and Overbalanced kalemia = kalemia was reversed at day 2 (from hypo to hyperkalemia or the opposite). | | | | | | |

### Additional Figure 1. Distribution of serum potassium levels at admission in the overall population


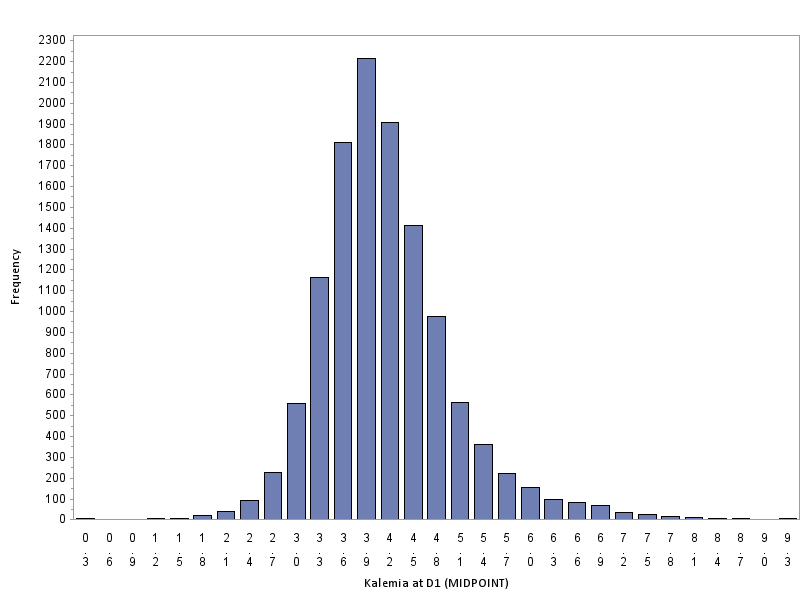


The number of patients

Serum potassium level (from 0.3 mmol/L to 9.3 mmol/L

*

### *Medical patients were more likely to have dyskalemia than surgical patients, p <0.01) Additional Figure 3. Prevalence of hypokalemia* according to the presence of underlying disease

*Chi square

*Hypokalemia = [K+] <3.5 mmol/L

Knaus Scale definitions were used to record pre-existing chronic organ failures, including respiratory, cardiac, hepatic, renal, and immunological dysfunctions [Knaus WA, Zimmerman JE, Wagner DP, et al.: APACHE-acute physiology and chronic health evaluation: a physiologically based classification system. *Crit Care Med* 1981; 9:591–597]

**Additional Figure 4. Prevalence of hyperkalemia* according to the presence of underlying disease**

*Hyperkalemia [K+] > 5 mmol/L

Knaus Scale definitions were used to record pre-existing chronic organ failures, including respiratory, cardiac, hepatic, renal, and immunological dysfunctions [Knaus WA, Zimmerman JE, Wagner DP, et al.: APACHE-acute physiology and chronic health evaluation: a physiologically based classification system. *Crit Care Med* 1981; 9:591–597]

### Additional Figure 5. Respiratory conditions in mild hyperkalemic patients (in red) in comparison to no-dyskaliemic patients (in black) and to other dyskaliemic patients (in blue) at admission

ARF: Acute Respiratory Failure; MV: Mechanical Ventilation; NVI: Non-invasive Ventilation; SOFA resp>2: points from respiratory item above
